# Does neurocognition predict personal recovery over time in psychotic disorder patients?

**DOI:** 10.1016/j.scog.2026.100425

**Published:** 2026-02-19

**Authors:** R. Rietveld, A.M. Kamperman, B.C. van Aken, G.H.M. Pijnenborg, C.L. Mulder

**Affiliations:** a Department of Psychiatry, Erasmus MC, the Netherlands; b Department of Psychotic Disorders, GGZ Drenthe, Assen, the Netherlands; c Department of Clinical and Developmental Neuropsychology, Faculty of Behavioral and Social Sciences, University of Groningen, Groningen, the Netherlands; d Parnassia Mental Health Care, Rotterdam, the Netherlands; e ESPRi - Epidemiological and Social Psychiatric Research Institute, Department of Psychiatry, Erasmus University Medical Center, Rotterdam, the Netherlands

**Keywords:** Neurocognition, Cognition, Recovery, Personal recovery, Psychotic disorders

## Abstract

**Background:**

Personal recovery has been recognized as an important goal in patients with psychotic disorders. It is defined as a deeply personal, unique process of living a satisfying life even with the limitations caused by the illness. Neurocognitive impairments are a core feature of psychotic disorders and proven to be a key determinant of functional outcomes, such as daily- and social functioning, work and independent living. However, the relationship with personal recovery remains unclear. Few studies investigated the relationship, and the findings remains inconclusive. This study explores the relationship between neurocognition and personal recovery, both cross-sectionally and over a year time.

**Method:**

Data from baseline and T1 (one year later) was used from the UP's cohort; this is a longitudinal observational study (*n* = 366) of schizophrenia spectrum disorder patients. Personal recovery was assessed using the ReQOL and the Individual Recovery Outcomes Counter (I.ROC). Neurocognition was assessed using the BACS (composite score and subdomains). Linear mixed models were used to analyze the association between neurocognition and personal recovery over time.

**Results:**

Global neurocognition and neurocognitive domains (verbal memory, working memory, motor speed, verbal fluency, attention and executive function) were not associated with personal recovery at baseline and after a one-year period. The models controlling for age and sex explained only a small proportion of the variance, adding the PANSS-R significantly improved the explained variance. Additional analyses showed robust finding across different personal recovery measures using the I.ROC. Personal recovery improved with 6.9% between baseline and one year later: the ReQOL improved from 57.6% to 64.5%, the I.ROC improved from 43.9% to 50.8%.

**Conclusion:**

Neurocognition did not predict personal recovery in this study. Patients with and without neurocognitive impairments showed similar levels of personal recovery at baseline and over a one-year time. This finding suggests that personal recovery can be accomplished in the presence of neurocognitive impairments and highlights the importance of addressing both domains independently. Psychotic symptoms are a stronger predictor of personal recovery than neurocognitive impairments. Future research is needed to investigate possible indirect relationships through metacognition.

## 1 Introduction

Over the past few decades, personal recovery has been recognized as an important goal in patients with severe mental illness, among others psychotic disorders (Leendertse et al., 2021; Mathew et al., 2023; van der Stel, 2012). In contrast to clinical recovery (reduction of symptoms), personal recovery entails a patient-centered view of recovery (van der Stel, 2012; van Weeghel et al., 2019). It is defined as “a deeply personal, unique, process of changing one's attitudes, values, feelings, goals, skills and/or roles. It is a way of living a satisfying, hopeful and contributing life even with the limitations caused by the illness” (Anthony, 1993). It involves changes in all for the individual relevant areas of life (van der Stel, 2012). Even though it is a highly personal process, a systematic review by Leamy et al. (2011) identified five key recovery processes summarized under the acronym CHIME: Connectedness, Hope, Identity, Meaning in Life and Empowerment. The CHIME framework is widely used and seems to be a fitting framework accounting for most personal recovery experiences (Penas et al., 2020; Stuart et al., 2017; Vogel et al., 2020). Using a meta-analytic approach of longitudinal studies in psychotic disorder patients, only modest changes in personal recovery have been observed, indicating substantial need for improvement (de Winter et al., 2024). Therefore, it is important to identify determinants of personal recovery.

Neurocognitive impairments could be one of those factors. These impairments have been established as a core feature of schizophrenia spectrum disorders (Bora et al., 2010; Gold and Harvey, 1993; Heinrichs and Zakzanis, 1998; Kahn and Keefe, 2013; Sheffield et al., 2018; Zanelli et al., 2019). Neurocognitive impairments are recognized as key determinants of functional outcomes such as daily functioning, occupational functioning, social functioning and independent living (Brekke et al., 2009; Fett et al., 2011; Green, 1996; Green et al., 2000; Harvey, 2014). However, their relationship with personal recovery remains unclear as the available evidence to this day is inconclusive. In a cross-sectional study, a positive association was found between specific neurocognitive domains (such as motor skills, executive function and verbal-linguistic neurocognition) and personal recovery in psychotic disorder patients? (Ritsner, 2007; Tan et al., 2020). On the other hand, in the same group of patients and also cross-sectionally, a negative association was found between global neurocognition and neurocognitive reasoning with personal recovery (Narvaez et al., 2008; Siu et al., 2015). An older meta-analysis found a positive relationship between letter fluency and personal recovery and a negative relationship between crystallized verbal skills and personal recovery, and no relationship for global neurocognition or other specific neurocognitive domains and personal recovery (Tolman and Kurtz, 2012). With regard to longitudinal designs, only two studies were identified: Prouteau et al. (2005) found a negative relationship between baseline sustained attention and personal recovery over time and Kurtz et al. (2012) found a positive relationship between baseline verbal memory and crystallized verbal skill with personal recovery over time. In these studies, varying ways of measuring personal recovery were used, as well as small sample sizes (*N* ≤ 62). A recent meta-analysis reported no association between neurocognition and personal recovery (de Winter et al., 2025). However, only one of the above-mentioned studies (Prouteau et al., 2005) was included in this meta-analysis based on the strict inclusion criteria.

Taken together, these studies indicate that there might be a relationship between (specific domains of) neurocognition and personal recovery in patients with a psychotic disorder, but this is not well established. The available evidence is based on varying personal recovery measures and mostly underpowered samples. This study aims to address these inconsistencies and limited evidence using a larger sample size, a well-defined and broad set of neurocognitive measures and personal recovery measures. In addition, our models will be adjusted for psychotic symptoms, as a potential confounding factor, since these symptoms have been shown to be associated with neurocognitive performance (de Winter et al., 2025). Through this study, we seek to provide more clarity and understanding of neurocognition as a possible determinant of personal recovery.

## 2 Methods

### 2.1 Procedure

The UP's study is an ongoing observational cohort of 366 patients intended to examine processes of recovery in people diagnosed with psychotic disorders over a 10-year period (Mulder et al., 2021; van Aken et al., 2021). Participants were, at time of inclusion, patients from mental health care institutions located in the southwestern Netherlands. Inclusion criteria were a primary diagnosis of a schizophrenia spectrum disorders according to a clinical diagnosis using the DSM-IV criteria and aged between 18 and 65. The only exclusion criterion was insufficient proficiency in Dutch. This study was approved by the Medical Ethical Review Board of Erasmus MC (study number NL58697.078.17). All participants gave informed consent. At the time of writing, all participants had completed T0 (baseline) and one year later (T1) and were therefore included in this study.

### 2.2 Measures

#### 2.2.1 Neurocognition: performance-based

For assessing performance-based neurocognition, we used the Brief Assessment of Cognition in Schizophrenia (BACS) test. The BACS was developed as a tool for measuring the six most impaired cognitive domains in schizophrenia (Keefe et al., 2004). These are: verbal memory, assessed with the List Learning task. Working memory is assessed with the Digit Sequencing task. Motor speed is assessed with the Token Motor task. Verbal fluency is assessed with the semantic- and letter fluency task. Attention and processing speed is assessed with the Symbol Coding task. Executive function is assessed with the Tower of London task. All tests are administered within their given time limit. For overall cognitive impairment, a composite score could be calculated using the six cognitive domains (Keefe et al., 2004). Single missing values were replaced by the series mean score. A higher score indicates better cognition. *Z*-scores were calculated based on both our study population, and on a general population based on the reference scores provided in the manual (Keefe et al., 2008). The ICC for the BACS ranged between 0.86 and 0.92 for patients, indicating a high reliability (Keefe et al., 2004). Our sample indicated good internal consistency (α =0.76).

#### 2.2.2 Personal recovery

Personal Recovery was assessed by using both the 10-item version of the Recovering of Quality of Life (ReQOL) questionnaire (Keetharuth et al., 2017) and the the Individual Recovery Outcomes Counter (I.ROC).

The ReQOL is a self-report questionnaire including 10 statements describing thoughts, feelings and activities over the past week. The answers are on a 5-point Likert scale with a range from “0” (None of the time) to “4” (most or all of the time). A total score can be calculated with a range from 0 to 40. A higher score indicates better personal recovery. The clinical range for the ReQOL is considered at a score of 24 and below (Keetharuth et al., 2017). In this study we used the ReQOL scores on both baseline and year 1. The Dutch version and our sample demonstrated good internal consistency (α = 0.94 and α =0.86, respectively) (van Aken et al., 2020).

The I.ROC will be used for an additional analysis to investigate the robustness of results with varying personal recovery measures. The I.ROC is a 12-item self-report questionnaire assessing different aspects of personal recovery over the past three months (Monger et al., 2013). It consists of four subscales (Home, Opportunity, People and Empowerment) with 3 questions each. The answers are on a 6-point Likert scale with a range from “1” (never) to “6” (always). The questionnaire is designed and proven to assess recovery in clinical practice (Ion et al., 2013). A total score can be calculated with a range from 12 to 72. A higher score indicates better personal recovery. The clinical range for the I.ROC is considered at a score of 51 and below (De Beurs et al., 2024). It demonstrated good internal consistency (α ranged from 0.86 to 0.88) (Monger et al., 2013; Sportel et al., 2023). Similar internal consistency was found in our sample (α =0.84). In this study we used the I.ROC total scores on both baseline and year 1.

### 2.3 Covariates

#### 2.3.1 Psychotic symptoms

The Positive and Negative Symptom Scale – Remission (PANSS-R) is a short version of the PANSS (Kay et al., 1987). The PANSS is used to measures remission of psychotic symptoms in the last two weeks. This version contains 8 of the original 30 items: 3 positive symptoms, 3 negative symptoms and 2 generic symptoms (Andreasen et al., 2005). Good internal consistency was demonstrated in earlier research and within our sample (α =0.85 (Sakinyte and Holmberg, 2023) and α =0.75, respectively). A higher score indicates higher severity of psychotic symptoms. For this study we used the PANSS-R scores at baseline.

#### 2.3.2 Employment status and living conditions

Studies showed that employment status and living conditions are associated with quality of life (Fujino et al., 2016; Hansson et al., 2002; Leendertse et al., 2021). Employment status is categorized into three groups: Paid employment, unpaid employment, unemployment and retirement. For the statistical analyses the group ‘retirement’ was excluded due to its small sample size (*N* = 2). Living conditions is categorized into three groups: independent living, living with parents/family and living at a healthcare institution.

### 2.4 Data analysis

The statistical analyses were performed in R studio (RStudio team, 2023), using packages: lme4 and nlme (Bates et al., 2015; Pinheiro et al., 2013). Descriptives and demographic variables were calculated and summarized. Pearson correlations were calculated between all measures and covariates. Linear mixed models were used to analyze the relationship between the BACS and ReQOL. Seven linear mixed models were fitted, using the BACS composite score and the six neurocognitive domains (verbal memory, digit sequencing, token motor, verbal fluency, symbol coding, tower of London) as separate predictors. In these models the fixed effects included age, sex, PANSS-R, employment, living conditions and time (baseline, year 1). The interaction between time and the respective BACS score were included to analyze the effect of BACS score on the ReQOL over time. The participants were entered as random effects to account for individual variability and for the dependence of test scores (Gurka et al., 2011). The covariates were added in a hierarchical manner (first age and sex, second PANSS-R, third employment and living conditions) to examine their unique contribution.

As additional analyses to investigate robustness of the findings across different personal recovery measures, all models will be replicated with the I.ROC as the dependent variable. The data of 267 participants was available at T1. To assess whether data were not missing at random, we performed independent sample *t*-tests between the non-responders and responders at T1 at the baseline measures and demographic variables used in this study. Results showed no indication for not missing at random. Assumptions were checked and the assumption of independence was violated for all models with a Durbin-Watson statistic of ~1.5, *p* < .05 (Durbin and Watson, 1992). Further investigation showed that the violation did not significantly influence the model, and we chose to proceed without any adjustments.

## 3 Results

### 3.1 Demographic variables study sample

The study sample consists of 366 participants with a mean age of 41.5 (12.5) years old, two-thirds were male (66%), and most common primary diagnoses was schizophrenia (42%).

**Fig. 1:**
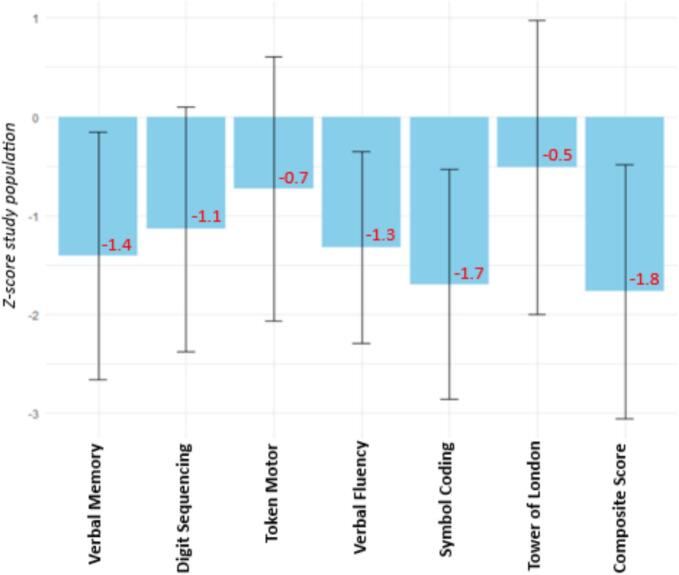
*Z*-scores and their standard deviation of the study population compared to general population reference scores (Keefe et al., 2008).

Mean BACS raw scores are shown in Table 1 and the BACS z-scores of our study population compared to general population reference scores are shown in Fig. 1. Compared to the general population, our participants scored on average lower on the composite score and all neurocognitive domains. Symbol Coding was the most impaired domain and Tower of London the least impaired domain. Notably, the variance of Token Motor task and Tower of London show substantial overlap with general population scores, meaning that in those two domains our participants show similar performance as the general population. Our participants scored noticeably better compared to an inpatient population (Haddad et al., 2021) and slightly to moderately better than outpatient populations (Araújo et al., 2015; Wang et al., 2016).

**Table 1:** Demographic and clinical variables of the study sample (*n* = 366).

		Mean (SD)	N (%)
Age		41.5 (12.5)	
Sex (female)			126 (34%)
Education level	No education completed		26 (7%)
	Primary school		64 (17%)
	Secondary school		153 (42%)
	Secondary vocational education		85 (23%)
	Higher education		38 (11%)
Diagnosis	Schizophrenia – 295.90		152 (42%)
	Schizoaffective – 295.70		35 (10%)
	Un- or otherwise specified psychotic spectrum disorder – 298.9		97 (26%)
	Brief psychotic disorder – 298.8		35 (10%)
	Other^a^		46 (12%)
Employment	Paid employment		70 (23%)
	Unpaid work		83 (27%)
	Unemployed		147 (49%)
	Retired		2 (1%)
Living situation	Independent living		225 (65%)
	With parents/family		51 (15%)
	(Mental) healthcare institution		68 (20%)
BACS^b^	Verbal Memory	34.8 (11.1)	
	Digit Sequencing	17.6 (4.7)	
	Token Motor	61.6 (17.6)	
	Verbal fluency	37.5 (11.1)	
	Symbol Coding	40.9 (13.1)	
	Tower of London	15.5 (4.8)	
	Composite Score	208.6 (42.8)	
PANSS-R		2.0 (0.8)	
REQOL	Baseline	25.7 (7.5)	
	Year 1	27.7 (7.8)	
I.ROC	Baseline	49.9 (10.0)	
	Year 1	51.8 (9.6)	

a Delusional disorder (*N* = 8), … induced by drugs/stress (N = 3), schizophreniform disorder (N = 2), Specific schizophrenia spectrum diagnosis unknown (*N* = 33).

b BACS raw scores.

The ReQOL total score at baseline was 25.7 (7.5), with 57.6% scoring in the non-clinical range (ReQOL >24). At year 1, the ReQOL total score was 27.7 (7.8), with 64.5% scoring in the non-clinical range. The I.ROC total score at baseline was 49.9 (10.0), with 43.9% scoring in the non-clinical range (I.ROC >51). At year 1, the I.ROC total score was 51.8 (9.6), with 50.8% scoring in the non-clinical range.

### 3.2 Correlations between neurocognition and personal recovery

**Fig. 2:**
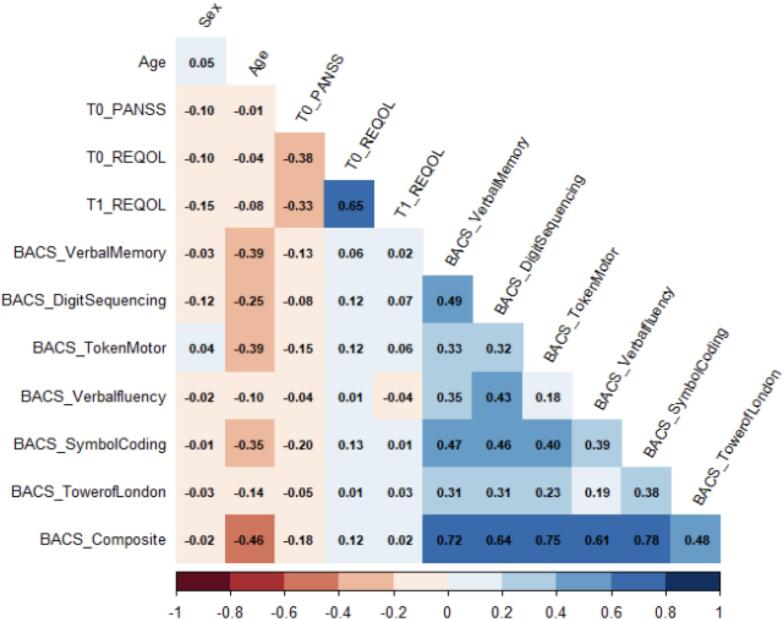
Correlations between de BACS, ReQOL, PANSS-R.

Fig. 2 shows the correlations among the BACS, ReQOL and the covariates. The ReQOL scores at baseline and year 1 demonstrated no significant correlation with the BACS scores (*r* = 0.01–0.14, *p* > .05). The PANSS-R demonstrated a significant moderate correlation with the ReQOL scores at baseline (*r* = −0.38, p < .05) and year 1 (*r* = −0.33, p < .05).

### 3.3 The association between neurocognition and personal recovery

Linear mixed models were calculated to examine the effect of the composite score and each of the six neurocognitive domains on the trajectory of personal recovery between baseline and year 1 (Table 2). Detailed information can be found in appendix A1.

**Table 2:** linear mixed models examining if neurocognition predicts the course of personal recovery.

	Fully adjusted models (age, sex, PANSS-R, employment, living condition)			
	Standardized estimate	*p*-Value	Marginal R^2^	Conditional R^2^
Time	0.10	*p* *<* *.001*	0.20	0.66
BACS – Composite	−0.02	*p* *=* *.91*		
Time ∗ BACS – Composite	−0.03	*p* *=* *.28*		
Time	0.11	*p* *<* *.001*	0.21	0.67
BACS – Verbal Memory	−0.03	*p* *=* *.61*		
Time ∗ Verbal Memory	0.00	*p* *=* *.87*		
Time	0.10	*p* *<* *.001*	0.21	0.68
Digit Sequencing	0.04	*p* *=* *.29*		
Time ∗ Digit Sequencing	−0.02	*p* *=* *.46*		
Time	0.11	*p* *<* *.001*	0.21	0.67
Token Motor	0.00	*p* *=* *.67*		
Time ∗ Token Motor	−0.02	*p* *=* *.52*		
Time	0.11	*p* *<* *.001*	0.21	0.67
Verbal Fluency	−0.04	*p* *=* *.93*		
Time ∗ Verbal Fluency	−0.04	*p* *=* *.16*		
Time	0.11	*p* *<* *.001*	0.21	0.66
Symbol Coding	0.00	*p* *=* *.65*		
Time ∗ Symbol Coding	−0.03	*p* *=* *.28*		
Time	0.10	*p* *<* *.00*	0.20	0.66
Tower of London	0.00	*p* *=* *.92*		
Time ∗ Tower of London	0.02	*p* *=* *.55*		

In the linear mixed models controlling for age and sex, neither the composite score nor the six neurocognitive domains showed significant associations with the ReQOL (β ranged from −0.02 to 0.09, *p* > .05) (Appendix A1). Similarly, none of the interaction effects between neurocognitive domain and time were statistically significant (β = −0.03–0.03, p > .05). The variance explained by the fixed effects (Marginal R^2^) by each model was either 3% or 4%, indicating that neurocognition, age and sex together explained only a small proportion of the variance.

In the linear mixed models controlling for age, sex and the PANSS-R, the main effects of the six neurocognitive domains and the composite score remained statistically non-significant (β ranged from −0.05 to 0.05, p > .05). Detailed estimates are presented in Appendix A1. Similarly, none of the interaction effects between main effects and time were statistically significant (β ranging from −0.03 to 0.02, p > .05). The variance explained by the fixed effects (Marginal R^2^) was 17 or 18%, indicating that the PANSS-R explained an additional 13 or 14% of the variance.

In the fully adjusted linear mixed models (controlling for age, sex, PANSS-R, employment and living conditions), the main effects of the six neurocognitive domains and the composite score remained statistically non-significant (β ranged from −0.04 to 0.04, p > .05) (Table 2). The variance explained by the fixed effects was 20 or 21%, indicating that employment and living conditions explained only a few additional percent of the variance.

Both the partially and fully adjusted models indicate that patients with different levels of cognitive impairment show comparable trajectories of personal recovery at baseline and over time.

### 3.4 Additional analyses – I.ROC questionnaire

As an additional analysis, the main analyses were replicated with the I.ROC questionnaire as outcome. Detailed information can be found in appendix A2. The ReQOL and I.ROC showed a strong correlation at baseline (*r* = 0.68, p < .001) and year 1 (*r* = 0.70, *p* < .001).

In the linear mixed models controlling for age and sex, the main effects of the composite score (β = 0.14, *p* < .01), Digit Sequencing (β = 0.11, p < .01), Token Motor (β = 0.13, p < .05), Symbol Coding (β = 0.12, *p* < .05) and Tower of London (β = 0.11, p < .05) were statistically significant (Appendix A2). None of the interaction effects between the main effects and time were statistically significant (β ranged from 0.00 to −0.05, p > .05). The variance explained by the fixed effects (Marginal R^2^) for each model was between 3 and 5%, consistent with the findings of the main analysis.

In the linear mixed models controlling for age, sex and the PANSS-R, only the main effects of Digit Sequencing (β = 0.08, p = .02) and Tower of London (β = 0.09, *p* < .05) remained statistically significant (Appendix A2). None of the interaction effects between the main effects and time were statistically significant (β ranged from 0.00 to −0.05, *p* > .05). The variance explained by the fixed effects (Marginal R^2^) by each model was 17% or 18%, consistent with the findings of the main analysis.

In the fully adjusted linear mixed models (controlling for age, sex, PANSS-R, employment status and living condition) the main effect of Digit Sequencing remained statistically significant (β = 0.09, *p* = .02) but the main effect of Tower of London was no longer statistically significant (β = 0.09, *p* = .07). The variance explained by the fixed effects (Marginal R^2^) by each model was between 18% and 20%, consistent with the findings of the main analysis.

## 4 Discussion

Our study aimed to investigate the cross-sectional and longitudinal relationship between neurocognition and personal recovery in patients with a psychotic disorder. The results showed that global neurocognition and specific neurocognitive domains (verbal memory, working memory, motor speed, verbal fluency, attention and executive function) were not associated with personal recovery at baseline and over a one-year period. Psychotic disorder patients with neurocognitive impairments experience similar levels of personal recovery as those without neurocognitive impairments. These findings suggest that personal recovery can be achieved in the presence of neurocognitive impairments, and both should be addressed in mental health care setting.

Our results partially align with previous findings, most of which reported no association between the majority of the neurocognitive domains and personal recovery (Kurtz et al., 2012; Narvaez et al., 2008; Prouteau et al., 2005; Ritsner, 2007; Tan et al., 2020; Tolman and Kurtz, 2012). However, verbal neurocognitive domains provided the most consistent evidence of an association with personal recovery in previous studies (Kurtz et al., 2012; Tan et al., 2020; Tolman and Kurtz, 2012). This inconsistency with our findings might be explained by the difference in personal recovery measures used. Most of the studies used measures focused on satisfaction in varying life domains (“how satisfied are you with your daily activities?”), commonly referred as subjective quality of life measures (s-QoL). In contrast, the questionnaires used in our study emphasized more on mental wellbeing and recovery (“I felt hopeful about my future”), which are classified as personal recovery measures. Our findings contribute to the notion that s-QoL and personal recovery are closely related yet represent distinct concepts. This is supported by the moderate correlation found between measures of s-QoL and personal recovery (Murphy et al., 2023), as well as two studies that found no association between neurocognition and personal recovery measures (Maas et al., 2024; Morrison et al., 2013). From a methodological viewpoint, the inconsistency between previous literature and our findings could be explained by the small sample sizes used (*N* ≤ 62) (Kurtz et al., 2012; Prouteau et al., 2005; Ritsner, 2007; Tan et al., 2020). Additionally, Tolman and Kurtz (2012) emphasized the preliminary nature of their meta-analysis given the limited number of studies, and noted that their findings should be interpreted with caution given the large variability across studies. The reported associations might therefore reflect spurious correlations rather than true underlying associations.

A more theoretical explanation is that, rather than the neurocognitive impairments itself, it is the capacity to cope with those neurocognitive impairments (e.g. through metacognition) that is associated with personal recovery. Metacognition - defined as the awareness of one's own thoughts and behaviors and the ability to therefore monitor and change behavior (Moritz and Lysaker, 2018) - has proven to be related to positive changes in patients' attitudes towards illness, increased sense of autonomy, self-esteem and setting meaningful goals (Lysaker et al., 2015; Moritz et al., 2018). All of which are key elements of personal recovery (Leamy et al., 2011). Future research is needed to confirm this.

When we repeated our analyses with the I.ROC as outcome, we found statistically significant main effects for Digit Sequencing and Tower of London in the fully adjusted models. This means that at baseline working memory and executive functioning are associated with personal recovery measured with the I.ROC. However, their effect sizes were small, and we did not correct for multiple testing. Furthermore, the effect sizes were well below the reliable change index for the I.ROC (RCI = 9.62) meaning that there is no (clinically) meaningful change (Jacobson and Truax, 1992). A lower percentage of patients were classified as personally recovered measured with the I.ROC compared to the ReQOL on both time-points, although both questionnaires showed similar improvement between baseline and one-year later (6.9%). This suggests that although both questionnaires show significant overlap and overall comparable results, they might capture slightly different aspects of personal recovery.

Another finding from our study is the weak association between neurocognition and psychotic symptoms, which supports the notion that neurocognition and psychotic symptoms are distinguishable symptom dimensions, and that psychotic symptoms are a stronger predictor of personal recovery than neurocognitive impairments (Altınbaş et al., 2020; Starzer et al., 2024).

Several limitations must be mentioned. First, as our study mainly consisted of chronic psychotic disorders, they might not be generalizable to different stages of the illness, such as a first episode patient population. Compared to first episode patients, a chronic population is more likely to have received interventions that might have mitigated the impact of neurocognitive impairment. Second, although this study had a longitudinal design, only data from T1 (after a year) was available at the time of writing. This provided limited insight in the relationship over longer periods, which is especially important since personal recovery is considered a process rather than an static outcome (Anthony, 1993; Leamy et al., 2011; van Weeghel et al., 2019). Thirdly, although we did not find a clear indication of selective drop-out (i.e. not missing at random), it should be noted that at year 1 data of the ReQOL was available of 267 participants out of the 366 due to that the participants actively declined, or we were not able to reach them for the interview. We did not collect data on the reasons why participants declined. Informally, some reported they did not want to revisit the topics related to their diagnosis both because they were doing well or because they were struggling. This could have influenced our results in either direction. Lastly, as data were not available on antipsychotic medication or substance abuse, we were unable to adjust for those variables in the analyses. Previous studies have shown that different types of antipsychotic medication (Awad and Voruganti, 2004; Crutzen et al., 2025) and substance abuse (Aras et al., 2013; Desalegn et al., 2020; Duke et al., 2001) are associated with quality of life which may influenced our results.

Our study highlights several strengths. To our knowledge this is one of the first studies with a large sample size to analyze this association. Additionally, the additional analyses with the I.ROC confirmed robustness of our findings across different measures of personal recovery. Even though the additional analyses with the I.ROC showed a small significant effect for the Tower of London, as no correction for multiple testing was applied it did not undermine the main analyses. Furthermore, the characteristics of our study population are comparable with the F-ACT population in the Netherlands (Kortrijk et al., 2019), reassuring the generalizability for this population. Lastly, the ReQOL and I.ROC are developed in collaboration with mental health service users and therefore assesses outcomes they consider most central to them in recovering their quality of life (Keetharuth et al., 2018; Monger et al., 2013). The ReQOL has even been recognized as an effective tool assessing personal recovery in psychotic- and personality disorders patients (McKenzie et al., 2022).

Future research should investigate whether there may be an indirect relationship between neurocognition and personal recovery through metacognition, as mentioned before. Other promising variables to investigate as potential mediators in this association are social cognition and negative symptoms, as previous studies found evidence that both mediate the association between neurocognition and functioning (Giordano et al., 2024; Schmidt et al., 2011). This raises the question if social cognition and negative symptoms play a similar role in the association between neurocognition and personal recovery. Furthermore, more research is needed to clarify the possible relationship between neurocognition (specifically the verbal neurocognitive domains) and s-QoL. Lastly, as a lot of variances of personal recovery remained unexplained, further research is essential to identify factors that facilitate or hinder personal recovery. For example, in this study we focused on performance-based neurocognition. However, neurocognition can in addition be assessed using self-report measures (Broadbent et al., 1982; Fisher et al., 2016; Rosa et al., 2013; Roth et al., 2014). Surprisingly, these measures also show no or weak correlations (Haugen et al., 2021; Nordvall et al., 2017; van Aken et al., 2023). It is thought that performance-based measures reflect someone's ability (skills) whereas self-report measures reflect what one actually does in the real world (performance) (Bowie and Harvey, 2006; Marcotte et al., 2022; van Aken et al., 2023). It therefore seems plausible that self-report measures are associated with personal recovery.

Based on our findings, both personal recovery and neurocognitive impairments should be addressed independently. Personal recovery, as this is one of the most important processes for patients, and neurocognitive impairments, given the well-known negative impact on multiple life domains. However, although the majority recognizes the need for targeted interventions for neurocognitive impairments, less than 50% of mental health care professionals reported giving little or no consideration to neurocognitive impairments. Several barriers are identified such as insufficient knowledge about neurocognitive impairments, lack of time and resources and patients who themselves are unaware of their cognitive difficulties (Agüera-Ortiz et al., 2025; Saperstein et al., 2021; Sumiyoshi et al., 2025). Another noteworthy barrier is that neurocognitive impairments are easily overlooked and misinterpreted as motivation problems while they in fact reflect an underlying inability (Palmisano et al., 2020). Taken together, this addresses the need for improved awareness of neurocognitive impairments among both mental health professionals as their patients and more time-effective instruments such as cognitive screeners (Stainton et al., 2025).

To conclude, our findings highlight that personal recovery can be accomplished in the presence of neurocognitive impairments, underscoring the importance of addressing both domains independently in treatment.

## Funding statement/disclosure

The authors report no conflicts with any product mentioned or concept discussed in this article.

Astrid Kamperman is funded by the Epidemiological and Social Psychiatric Research Institute (ESPRi), a consortium of academic and non-academic research groups at the following institutes of mental health care (GGz): Antes Parnassia Group, Pameijer, GGz Breburg, GGz Delfland, GGz Westelijk Noord-Brabant, Emergis and Yulius.

## CRediT authorship contribution statement

**R. Rietveld:** Writing – original draft, Visualization, Software, Project administration, Methodology, Formal analysis, Data curation, Conceptualization. **A.M. Kamperman:** Writing – review & editing, Supervision, Funding acquisition, Conceptualization. **B.C. van Aken:** Writing – review & editing, Supervision, Conceptualization. **G.H.M. Pijnenborg:** Writing – review & editing, Supervision, Conceptualization. **C.L. Mulder:** Writing – review & editing, Supervision, Funding acquisition, Conceptualization.

## Declaration of competing interest

The authors declare that they have no known competing financial interests or personal relationships that could have appeared to influence the work reported in this paper.

## Appendix A Supplementary data

Supplementary material Image 1

## References

[bb0005] Agüera-Ortiz L., Aragonés E., Buch-Vicente B., Mendive J.M., Peña M., Vieta E. (2025). Cognitive symptoms in schizophrenia: an analysis of awareness, assessment, and management practices among psychiatrists and primary care physicians. Front. Psych..

[bb0010] Altınbaş K., Guloksuz S., van Os J. (2020). Psychotic Disorders*:* Comprehensive Conceptualization and Treatments.

[bb0015] Andreasen N.C., Jr. W.T.C., Kane J.M., Lasser R.A., Marder S.R., Weinberger D.R. (2005). Remission in schizophrenia: proposed criteria and rationale for consensus. Am. J. Psychiatry.

[bb0020] Anthony W.A. (1993). Recovery from mental illness: the guiding vision of the mental health service system in the 1990s. Psychosocial Rehab. J..

[bb0025] Aras H.I., Yazar M.S., Altinbas K. (2013). Quality of life among dually diagnosed and non-substance-using male schizophrenia outpatients. S. Afr. J. Psychiatry.

[bb0030] Araújo G.E., de Resende C.B., Cardoso A.C.A., Teixeira A.L., Keefe R.S.E., Salgado J.V. (2015). Validity and reliability of the Brazilian Portuguese version of the BACS (Brief Assessment of Cognition in Schizophrenia). Clinics.

[bb0035] Awad A.G., Voruganti L.N. (2004). New antipsychotics, compliance, quality of life, and subjective tolerability—are patients better off?. Can. J. Psychiatry.

[bb0040] Bates D., Mächler M., Bolker B., Walker S. (2015). Fitting linear mixed-effects models using lme4. J. Stat. Softw..

[bb0045] Bora E., Yucel M., Pantelis C. (2010). Cognitive impairment in schizophrenia and affective psychoses: implications for DSM-V criteria and beyond. Schizophr. Bull..

[bb0050] Bowie C.R., Harvey P.D. (2006). Cognitive deficits and functional outcome in schizophrenia. Neuropsychiatr. Dis. Treat..

[bb0055] Brekke J.S., Hoe M., Green M.F. (2009). Neurocognitive change, functional change and service intensity during community-based psychosocial rehabilitation for schizophrenia. Psychol. Med..

[bb0060] Broadbent D.E., Cooper P.F., FitzGerald P., Parkes K.R. (1982). The cognitive failures questionnaire (CFQ) and its correlates. Br. J. Clin. Psychol..

[bb0065] Crutzen S., Gangadin S., Hua K.H., Veling W., Castelein S. (2025). The association of antipsychotic treatment and side effects with societal recovery and happiness: a naturalistic cohort study of people in long term care for a psychotic disorder. Eur. Psychiatry.

[bb0070] De Beurs E., Metz M.J., Nahar-van Venrooij L.M.W. (2024). Tijdschrift voor Psychiatrie(2018/2018).

[bb0075] de Winter L., Jelsma A., Vermeulen J.M., van Weeghel J., Hasson-Ohayon I., Mulder C.L., Boonstra N., Veling W., de Haan L. (2024). Long-term changes in personal recovery and quality of life among patients with schizophrenia spectrum disorders and different durations of illness: a meta-analysis. Schizophr. Bull..

[bb0080] de Winter L., Jelsma A., Vermeulen J.M., Vellinga A., van Weeghel J., Hasson-Ohayon I., Mulder C.L., Boonstra N., Veling W., de Haan L. (2025). Interrelationships of changes in outcome domains in patients with schizophrenia spectrum disorders: a meta-analysis. Acta Psychiatr..

[bb0085] Desalegn D., Girma S., Abdeta T. (2020). Quality of life and its association with current substance use, medication non-adherence and clinical factors of people with schizophrenia in Southwest Ethiopia: a hospital-based cross-sectional study. Health Qual. Life Outcomes.

[bb0090] Duke P.J., Pantelis C., McPhillips M.A., Barnes T.R. (2001). Comorbid non-alcohol substance misuse among people with schizophrenia: epidemiological study in central London. Br. J. Psychiatry.

[bb0095] Durbin J., Watson G.S. (1992).

[bb0100] Fett A.K., Viechtbauer W., Dominguez M.D., Penn D.L., van Os J., Krabbendam L. (2011). The relationship between neurocognition and social cognition with functional outcomes in schizophrenia: a meta-analysis. Neurosci. Biobehav. Rev..

[bb0105] Fisher C.A., Goodall J., Simmons M.B., Allott K., Hetrick S.E. (2016). Subjective ratings of neurocognitive functioning in depressed young people undergoing treatment: utility of a brief screening tool. Early Interv. Psychiatry.

[bb0110] Fujino H., Sumiyoshi C., Sumiyoshi T., Yasuda Y., Yamamori H., Ohi K., Fujimoto M., Hashimoto R., Takeda M., Imura O. (2016). Predicting employment status and subjective quality of life in patients with schizophrenia. Schizophr. Res. Cogn..

[bb0115] Giordano G.M., Pezzella P., Mucci A., Austin S.F., Erfurth A., Glenthøj B., Hofer A., Hubenak J., Libiger J., Melle I. (2024). Negative symptoms and social cognition as mediators of the relationship between neurocognition and functional outcome in schizophrenia. Front. Psych..

[bb0120] Gold J.M., Harvey P.D. (1993). Cognitive deficits in schizophrenia. Psychiatr. Clin. North Am..

[bb0125] Green M.F. (1996). What are the functional consequences of neurocognitive deficits in schizophrenia. Am. J. Psychiatry.

[bb0130] Green M.F., Kern R.S., Braff D.L., Mintz J. (2000). Neurocognitive deficits and functional outcome in schizophrenia: are we measuring the “right stuff”?. Schizophr. Bull..

[bb0135] Gurka M.J., Edwards L.J., Muller K.E. (2011). Avoiding bias in mixed model inference for fixed effects. Stat. Med..

[bb0140] Haddad C., Salameh P., Hallit S., Obeid S., Haddad G., Clément J.-P., Calvet B. (2021). Cross-cultural adaptation and validation of the Arabic version of the BACS scale (the brief assessment of cognition in schizophrenia) among chronic schizophrenic inpatients. BMC Psychiatry.

[bb0145] Hansson L., Middelboe T., Sørgaard K., Bengtsson-Tops A., Bjarnason O., Merinder L., Nilsson L., Sandlund M., Korkeila J., Vinding H. (2002). Living situation, subjective quality of life and social network among individuals with schizophrenia living in community settings. Acta Psychiatr. Scand..

[bb0150] Harvey P.D. (2014). What is the evidence for changes in cognition and functioning over the lifespan in patients with schizophrenia?. J. Clin. Psychiatry.

[bb0155] Haugen I., Stubberud J., Ueland T., Haug E., Øie M.G. (2021). Executive dysfunction in schizophrenia: predictors of the discrepancy between subjective and objective measures. Schizophr. Res. Cogn..

[bb0160] Heinrichs R.W., Zakzanis K.K. (1998). Neurocognitive deficit in schizophrenia: a quantitative review of the evidence. Neuropsychology.

[bb0165] Ion R., Monger B., Hardie S., Henderson N., Cumming J. (2013). A tool to measure progress and outcome in recovery. Brit. J. Mental Health Nurs..

[bb0170] Jacobson N.S., Truax P. (1992).

[bb0175] Kahn R.S., Keefe R.S. (2013). Schizophrenia is a cognitive illness: time for a change in focus. JAMA Psychiatry.

[bb0180] Kay S.R., Fiszbein A., Opler L.A. (1987). The positive and negative syndrome scale (PANSS) for schizophrenia. Schizophr. Bull..

[bb0185] Keefe R.S., Goldberg T.E., Harvey P.D., Gold J.M., Poe M.P., Coughenour L. (2004). The Brief Assessment of Cognition in Schizophrenia: reliability, sensitivity, and comparison with a standard neurocognitive battery. Schizophr. Res..

[bb0190] Keefe R.S.E., Harvey P.D., Goldberg T.E., Gold J.M., Walker T.M., Kennel C., Hawkins K. (2008). Norms and standardization of the Brief Assessment of Cognition in Schizophrenia (BACS). Schizophr. Res..

[bb0195] Keetharuth A.D., Brazier J., Connell J., Carlton J., Taylor Buck E., Ricketts T. (2017).

[bb0200] Keetharuth A.D., Brazier J., Connell J., Bjorner J.B., Carlton J., Buck E.T., Ricketts T., McKendrick K., Browne J., Croudace T. (2018). Recovering Quality of Life (ReQoL): a new generic self-reported outcome measure for use with people experiencing mental health difficulties. Br. J. Psychiatry.

[bb0205] Kortrijk H., Schaefer B., van Weeghel J., Mulder C.L., Kamperman A. (2019). Trajectories of patients with severe mental illness in two-year contact with flexible assertive community treatment teams using routine outcome monitoring data: an observational study. PloS One.

[bb0210] Kurtz M.M., Bronfeld M., Rose J. (2012). Cognitive and social cognitive predictors of change in objective versus subjective quality-of-life in rehabilitation for schizophrenia. Psychiatry Res..

[bb0215] Leamy M., Bird V., Le Boutillier C., Williams J., Slade M. (2011). Conceptual framework for personal recovery in mental health: systematic review and narrative synthesis. Br. J. Psychiatry..

[bb0220] Leendertse J.C.P., Wierdsma A.I., van den Berg D., Ruissen A.M., Slade M., Castelein S., Mulder C.L. (2021). Personal recovery in people with a psychotic disorder: a systematic review and meta-analysis of associated factors. Front. Psych..

[bb0225] Lysaker P.H., Kukla M., Belanger E., White D.A., Buck K.D., Luther L., Firmin R.L., Leonhardt B. (2015). Individual psychotherapy and changes in self-experience in schizophrenia: a qualitative comparison of patients in metacognitively focused and supportive psychotherapy. Psychiatry.

[bb0230] Maas I.L., Bohlken M.M., Gangadin S.S., Rosema B.-S., Veling W., Boonstra N., de Haan L., Begemann M.J.H., Koops S., Hamlett, Consortium O. (2024). Personal recovery in first-episode psychosis: beyond clinical and functional recovery. Schizophr. Res..

[bb0235] Marcotte T.D., Schmitter-Edgecombe M., Grant I. (2022).

[bb0240] Mathew S.T., Nirmala B.P., Kommu J.V.S. (2023). Personal meaning of recovery among persons with schizophrenia. Int. J. Soc. Psychiatry.

[bb0245] McKenzie E., Matkin L., Sousa Fialho L., Emelurumonye I.N., Gintner T., Ilesanmi C., Jagger B., Quinney S., Anderson E., Baandrup L. (2022). Developing an international standard set of patient-reported outcome measures for psychotic disorders. Psychiatr. Serv..

[bb0250] Monger B., Hardie S.M., Ion R., Cumming J., Henderson N. (2013). The Individual Recovery Outcomes Counter: preliminary validation of a personal recovery measure. Psychiatrist.

[bb0255] Moritz S., Lysaker P.H. (2018). Metacognition–what did James H. Flavell really say and the implications for the conceptualization and design of metacognitive interventions. Schizophr. Res..

[bb0260] Moritz S., Mahlke C.I., Westermann S., Ruppelt F., Lysaker P.H., Bock T., Andreou C. (2018). Embracing psychosis: a cognitive insight intervention improves personal narratives and meaning-making in patients with schizophrenia. Schizophr. Bull..

[bb0265] Morrison A.P., Shryane N., Beck R., Heffernan S., Law H., McCusker M., Bentall R.P. (2013). Psychosocial and neuropsychiatric predictors of subjective recovery from psychosis. Psychiatry Res..

[bb0270] Mulder C.L., van Aken B.C., Wierdsma A.I. (2021). Recovery in psychotic disorder patients: towards an integrative perspective [short communication]. Clin. Psychiatry.

[bb0275] Murphy O., Looney K., McNulty M., O’Reilly G. (2023). Exploring the factors that predict quality of life, and the relationship between recovery orientation and quality of life in adults with severe mental health difficulties. Curr. Psychol..

[bb0280] Narvaez J.M., Twamley E.W., McKibbin C.L., Heaton R.K., Patterson T.L. (2008). Subjective and objective quality of life in schizophrenia. Schizophr. Res..

[bb0285] Nordvall O., Jonsson B., Neely A.S. (2017). Self-reported and performance-based measures of executive functions in interned youth. Psychol. Crime Law.

[bb0290] Palmisano S., Fasotti L., Bertens D. (2020). Neurobehavioral initiation and motivation problems after acquired brain injury. Front. Neurol..

[bb0295] Penas P., Uriarte J.J., Gorbena S., Moreno-Calvete M.C., Ridgway P., Iraurgi I. (2020). Psychometric adequacy of recovery enhancing environment (REE) measure: CHIME framework as a theory base for a recovery measure. Front. Psych..

[bb0300] Pinheiro J.C., Bates D.M., DebRoy S., Sarkar D., Team R.C. (2013).

[bb0305] Prouteau A., Verdoux H., Briand C., Lesage A., Lalonde P., Nicole L., Reinharz D., Stip E. (2005). Cognitive predictors of psychosocial functioning outcome in schizophrenia: a follow-up study of subjects participating in a rehabilitation program. Schizophr. Res..

[bb0310] Ritsner M.S. (2007). Predicting quality of life impairment in chronic schizophrenia from cognitive variables. Qual. Life Res..

[bb0315] Rosa A.R., Mercadé C., Sánchez-Moreno J., Solé B., Bonnin C.D.M., Torrent C., Grande I., Sugranyes G., Popovic D., Salamero M. (2013). Validity and reliability of a rating scale on subjective cognitive deficits in bipolar disorder (COBRA). J. Affect. Disord..

[bb0320] Roth R.M., Isquith P.K., Gioia G.A., Goldstein S., Naglieri J.A. (2014). Handbook of Executive Functioning.

[bb0325] RStudio team (2023).

[bb0330] Sakinyte K., Holmberg C. (2023). Psychometric and clinical evaluation of schizophrenia remission criteria in outpatients with psychotic disorders. BMC Psychiatry.

[bb0335] Saperstein A.M., Medalia A., Bello I., Dixon L.B. (2021). Addressing cognitive health in coordinated specialty care for early psychosis: real-world perspectives. Early Interv. Psychiatry.

[bb0340] Schmidt S.J., Mueller D.R., Roder V. (2011). Social cognition as a mediator variable between neurocognition and functional outcome in schizophrenia: empirical review and new results by structural equation modeling. Schizophr. Bull..

[bb0345] Sheffield J.M., Karcher N.R., Barch D.M. (2018). Cognitive deficits in psychotic disorders: a lifespan perspective. Neuropsychol. Rev..

[bb0350] Siu C.O., Harvey P.D., Agid O., Waye M., Brambilla C., Choi W.K., Remington G. (2015). Insight and subjective measures of quality of life in chronic schizophrenia. Schizophr. Res. Cogn..

[bb0355] Sportel B.E., Aardema H., Boonstra N., Arends J., Rudd B., Metz M.J., Castelein S., Pijnenborg G.H.M. (2023). Measuring recovery in participants with a schizophrenia spectrum disorder: validation of the Individual Recovery Outcomes Counter (I. ROC). BMC Psychiatry.

[bb0360] Stainton A., Bryce S., Rattray A., Pert A., Zbukvic I., Fisher E., Anderson D., Bowden S.C., Chakma S., Cheng N. (2025). Validating cognitive screening in young people with first-episode psychosis: the CogScreen protocol. Early Interv. Psychiatry.

[bb0365] Starzer M., Hansen H.G., Hjorthøj C., Albert N., Lewandowski K.E., Glenthøj L.B., Nordentoft M. (2024). 20-year neurocognitive development following a schizophrenia spectrum disorder and associations with symptom severity and functional outcomes. Psychol. Med..

[bb0370] Stuart S.R., Tansey L., Quayle E. (2017). What we talk about when we talk about recovery: a systematic review and best-fit framework synthesis of qualitative literature. J. Ment. Health.

[bb0375] Sumiyoshi T., Ikezawa S., Inaba K., Marumoto T., Kusumi I., Nakagome K. (2025). Awareness and management of cognitive impairment associated with schizophrenia in psychiatrists and patients: results from a cross-sectional survey. Schizophr. Res. Cogn..

[bb0380] Tan E.J., Rossell S.L., Lee S.J. (2020). Impaired meaning-based cognitive skills are specifically associated with poorer subjective quality of life in schizophrenia. Personal. Med. Psychiatry.

[bb0385] Tolman A.W., Kurtz M.M. (2012). Neurocognitive predictors of objective and subjective quality of life in individuals with schizophrenia: a meta-analytic investigation. Schizophr. Bull..

[bb0390] van Aken B.C., de Beurs E., Mulder C.L., van der Feltz-Cornelis C.M. (2020). The Dutch Recovering Quality of Life questionnaire (ReQoL) and its psychometric qualities. Eur. J. Psychiatry.

[bb0395] van Aken B.C., Bakia A., Wierdsma A.I., Voskes Y., Van Weeghel J., van Bussel E.M.M., Hagestein C., Ruissen A.M., Leendertse P., Sewbalak W.V., van der Draai D.A., Hammink A., Mandos M.E., van der Gaag M., Bonebakker A.E., van der Feltz-Cornelis C.M., Mulder C.L. (2021). UP’S: A cohort study on recovery in psychotic disorder patients: design protocol. Front. Psych..

[bb0400] van Aken B., Rietveld R., Wierdsma A., Voskes Y., Pijnenborg G., van Weeghel J., Mulder C. (2023). Self-report versus performance based executive functioning in people with psychotic disorders. Schizophr. Res. Cogn..

[bb0405] van der Stel J.C. (2012).

[bb0410] van Weeghel J., van Zelst C., Boertien D., Hasson-Ohayon I. (2019). Conceptualizations, assessments, and implications of personal recovery in mental illness: a scoping review of systematic reviews and meta-analyses. Psychiatr. Rehabil. J..

[bb0415] Vogel J.S., Bruins J., Halbersma L., Lieben R.J., de Jong S., van der Gaag M., Castelein S. (2020). Measuring personal recovery in people with a psychotic disorder based on CHIME: a comparison of three validated measures. Int. J. Ment. Health Nurs..

[bb0420] Wang L.-J., Lin P.-Y., Lee Y., Huang Y.-C., Hsu S.-T., Hung C.-F., Chen C.-K., Chen Y.-C., Wang Y.-L., Tsai M.-C. (2016). Validation of the Chinese version of Brief Assessment of Cognition in Schizophrenia. Neuropsychiatr. Dis. Treat..

[bb0425] Zanelli J., Mollon J., Sandin S., Morgan C., Dazzan P., Pilecka I., Reis Marques T., David A.S., Morgan K., Fearon P., Doody G.A., Jones P.B., Murray R.M., Reichenberg A. (2019). Cognitive change in schizophrenia and other psychoses in the decade following the first episode. Am. J. Psychiatry.

