# Supplementary material for: Does neurocognition predict personal recovery over time in psychotic disorder patients?
Source: Schizophr Res Cogn. 2026 Feb 19;44:100425. doi: 10.1016/j.scog.2026.100425 (PMC12934219; doi:10.1016/j.scog.2026.100425)
Supplement: Supplementary file 1 — Supplementary material [file mmc1.docx]

**Appendix A1**

| Table 1: linear mixed model (adjusted for age, sex, PANSS-R, living conditions, employment situation) examining if neurocognition composite predicts the course of the ReQOL | | | | | | | | | | | | |
| --- | --- | --- | --- | --- | --- | --- | --- | --- | --- | --- | --- | --- |
|  | Model adjusted for age, sex | | | | Model adjusted for age, sex, PANSS | | | | Model adjusted for age, sex, PANSS, employment, living conditions | | | |
|  | SE | p-value | Marg. R^2^ | Cond. R^2^ | SE | p-value | Marg. R^2^ | Cond. R^2^ | SE | p-value | Marg. R^2^ | Cond. R^2^ |
| Time | .12 | p<.001 | .04 | .66 | .12 | p<.001 | .18 | .67 | .10 | p<.001 | .20 | .66 |
| Composite | .07 | p=.12 |  |  | .00 | p=.75 |  |  | -.02 | p=.91 |  |  |
| Time * Composite | -.03 | p=.35 |  |  | -.02 | p=.45 |  |  | -.03 | p=.28 |  |  |
| Age | -.03 | p=.35 |  |  | -.07 | p=.20 |  |  | -.10 | p=.13 |  |  |
| Sex (female) | -.24 | p=.09 |  |  | -.27 | p=.01 |  |  | -.23 | p=.04 |  |  |
| PANSS-R |  |  |  |  | -.39 | p<.001 |  |  | -.42 | p<.001 |  |  |
| Living indepent (ref) |  |  |  |  |  |  |  |  |  |  |  |  |
| With parents/family |  |  |  |  |  |  |  |  | -.26 | p=.13 |  |  |
| Healthcare institution |  |  |  |  |  |  |  |  | .11 | p=.45 |  |  |
| Employed (ref) |  |  |  |  |  |  |  |  |  |  |  |  |
| Unpaid employment |  |  |  |  |  |  |  |  | -.36 | p=.01 |  |  |
| Unemployed |  |  |  |  |  |  |  |  | -.21 | p=.11 |  |  |

| Table 2: linear mixed model (adjusted for age, sex, PANSS-R, living conditions, employment situation) examining if Verbal Memory predicts the course of the ReQOL | | | | | | | | | | | | |
| --- | --- | --- | --- | --- | --- | --- | --- | --- | --- | --- | --- | --- |
|  | Model adjusted for age, sex | | | | Model adjusted for age, sex, PANSS | | | | Model adjusted for age, sex, PANSS, employment, living conditions | | | |
|  | SE | p-value | Marg. R^2^ | Cond. R^2^ | SE | p-value | Marg. R^2^ | Cond. R^2^ | SE | p-value | Marg. R^2^ | Cond. R^2^ |
| Time | .11 | p<.001 | .03 | .67 | .12 | p<.001 | .17 | .66 | .11 | p<.001 | .21 | .67 |
| Verbal Memory | .03 | p=.60 |  |  | -.02 | p=.68 |  |  | -.03 | p=.61 |  |  |
| Time * Verbal Memory | .00 | p=.93 |  |  | .00 | p=.86 |  |  | .00 | p=.87 |  |  |
| Age | -.07 | p=.26 |  |  | -.08 | p=.13 |  |  | -.11 | p=.08 |  |  |
| Sex (female) | -.23 | p=.04 |  |  | -.28 | p<.01 |  |  | -.25 | p=.02 |  |  |
| PANSS-R |  |  |  |  | -.38 | p<.001 |  |  | -.43 | p<.001 |  |  |
| Living indepent (ref) |  |  |  |  |  |  |  |  |  |  |  |  |
| With parents/family |  |  |  |  |  |  |  |  | -.33 | p=.05 |  |  |
| Healthcare institution |  |  |  |  |  |  |  |  | .08 | p=.58 |  |  |
| Employed (ref) |  |  |  |  |  |  |  |  |  |  |  |  |
| Unpaid employment |  |  |  |  |  |  |  |  | -.38 | p<.01 |  |  |
| Unemployed |  |  |  |  |  |  |  |  | -.21 | p=.12 |  |  |

| Table 3: linear mixed model (adjusted for age, sex, PANSS-R, living conditions, employment situation) examining if Digit Sequencing predicts the course of the ReQOL | | | | | | | | | | | | |
| --- | --- | --- | --- | --- | --- | --- | --- | --- | --- | --- | --- | --- |
|  | Model adjusted for age, sex | | | | Model adjusted for age, sex, PANSS | | | | Model adjusted for age, sex, PANSS, employment, living conditions | | | |
|  | SE | p-value | Marg. R^2^ | Cond. R^2^ | SE | p-value | Marg. R^2^ | Cond. R^2^ | SE | p-value | Marg. R^2^ | Cond. R^2^ |
| Time | .11 | p<.001 | .04 | .69 | .11 | p<.001 | .17 | .66 | .10 | p<.001 | .21 | .68 |
| Digit Sequencing | .06 | p=.20 |  |  | .05 | p=.29 |  |  | .04 | p=.29 |  |  |
| Time * Digit Sequencing | -.01 | p=.63 |  |  | .00 | p=.73 |  |  | -.02 | p=.46 |  |  |
| Age | -.06 | p=.31 |  |  | -.04 | p=.20 |  |  | -.10 | p=.12 |  |  |
| Sex (female) | -.22 | p<.05 |  |  | -.06 | p=.01 |  |  | -.24 | p<.04 |  |  |
| PANSS-R |  |  |  |  | -.38 | p<.001 |  |  | -.43 | p<.001 |  |  |
| Living indepent (ref) |  |  |  |  |  |  |  |  |  |  |  |  |
| With parents/family |  |  |  |  |  |  |  |  | -.30 | p=.08 |  |  |
| Healthcare institution |  |  |  |  |  |  |  |  | .13 | p=.40 |  |  |
| Employed (ref) |  |  |  |  |  |  |  |  |  |  |  |  |
| Unpaid employment |  |  |  |  |  |  |  |  | -.35 | p<.02 |  |  |
| Unemployed |  |  |  |  |  |  |  |  | -.17 | p=.22 |  |  |

| Table 4: linear mixed model (adjusted for age, sex, PANSS-R, living conditions, employment situation) examining if Token Motor predicts the course of the ReQOL | | | | | | | | | | | | |
| --- | --- | --- | --- | --- | --- | --- | --- | --- | --- | --- | --- | --- |
|  | Model adjusted for age, sex | | | | Model adjusted for age, sex, PANSS | | | | Model adjusted for age, sex, PANSS, employment, living conditions | | | |
| n | SE | p-value | Marg. R^2^ | Cond. R^2^ | SE | p-value | Marg. R^2^ | Cond. R^2^ | SE | p-value | Marg. R^2^ | Cond. R^2^ |
| Time | .12 | p<.001 | .03 | .66 | .12 | p=<.001 | .17 | .68 | .11 | p<.001 | .21 | .67 |
| Token Motor | .09 | p=.07 |  |  | .04 | p=.39 |  |  | .00 | p=.67 |  |  |
| Time * Token Motor | -.02 | p=.57 |  |  | -.00 | p=.73 |  |  | -.02 | p=.52 |  |  |
| Age | -.02 | p=.74 |  |  | -.04 | p=.27 |  |  | -.10 | p=.13 |  |  |
| Sex (female) | -.21 | p=.06 |  |  | -.29 | p<.01 |  |  | -.26 | p=.02 |  |  |
| PANSS-R |  |  |  |  | -.38 | p<.001 |  |  | -.43 | p<.001 |  |  |
| Living indepent (ref) |  |  |  |  |  |  |  |  |  |  |  |  |
| With parents/family |  |  |  |  |  |  |  |  | -.29 | p=.09 |  |  |
| Healthcare institution |  |  |  |  |  |  |  |  | .11 | p=.47 |  |  |
| Employed (ref) |  |  |  |  |  |  |  |  |  |  |  |  |
| Unpaid employment |  |  |  |  |  |  |  |  | -.35 | p=.01 |  |  |
| Unemployed |  |  |  |  |  |  |  |  | -.22 | p=.10 |  |  |

| Table 5: linear mixed model (adjusted for age, sex, PANSS-R, living conditions, employment situation) examining if Verbal Fluency predicts the course of the ReQOL | | | | | | | | | | | | |
| --- | --- | --- | --- | --- | --- | --- | --- | --- | --- | --- | --- | --- |
|  | Model adjusted for age, sex | | | | Model adjusted for age, sex, PANSS | | | | Model adjusted for age, sex, PANSS, employment, living conditions | | | |
|  | SE | p-value | Marg. R^2^ | Cond. R^2^ | SE | p-value | Marg. R^2^ | Cond. R^2^ | SE | p-value | Marg. R^2^ | Cond. R^2^ |
| Time | .12 | p<.001 | .03 | .67 | .12 | p<.001 | .17 | .67 | .11 | p<.001 | .21 | .67 |
| Verbal Fluency | -.02 | p=.97 |  |  | -.05 | p=.67 |  |  | -.04 | p=.93 |  |  |
| Time * Verbal Fluency | -.03 | p=.31 |  |  | -.03 | p=.28 |  |  | -.04 | p=.16 |  |  |
| Age | -.05 | p=.37 |  |  | -.07 | p=.16 |  |  | -.10 | p=.08 |  |  |
| Sex (female) | -.23 | p=.03 |  |  | -.29 | p<.01 |  |  | -.27 | p=.01 |  |  |
| PANSS-R |  |  |  |  | -.38 | p<.001 |  |  | -.43 | p<.001 |  |  |
| Living indepent (ref) |  |  |  |  |  |  |  |  |  |  |  |  |
| With parents/family |  |  |  |  |  |  |  |  | -.32 | p=.06 |  |  |
| Healthcare institution |  |  |  |  |  |  |  |  | .11 | p=.46 |  |  |
| Employed (ref) |  |  |  |  |  |  |  |  |  |  |  |  |
| Unpaid employment |  |  |  |  |  |  |  |  | -.37 | p<.01 |  |  |
| Unemployed |  |  |  |  |  |  |  |  | -.21 | p=.11 |  |  |

| Table 6: linear mixed model (adjusted for age, sex, PANSS-R, living conditions, employment situation) examining if Symbol Coding predicts the course of the ReQOL | | | | | | | | | | | | |
| --- | --- | --- | --- | --- | --- | --- | --- | --- | --- | --- | --- | --- |
|  | Model adjusted for age, sex | | | | Model adjusted for age, sex, PANSS | | | | Model adjusted for age, sex, PANSS, employment, living conditions | | | |
|  | SE | p-value | Marg. R^2^ | Cond. R^2^ | SE | p-value | Marg. R^2^ | Cond. R^2^ | SE | p-value | Marg. R^2^ | Cond. R^2^ |
| Time | .12 | p<.001 | .04 | .67 | .12 | p<.001 | .18 | .66 | .11 | p<.001 | .21 | .66 |
| Symbol Coding | .08 | p=.06 |  |  | .00 | p=.68 |  |  | .00 | p=.65 |  |  |
| Time * Symbol Coding | -.04 | p=.15 |  |  | -.03 | p=.24 |  |  | -.03 | p=.28 |  |  |
| Age | -.04 | p=.45 |  |  | -.09 | p=.10 |  |  | -.10 | p=.12 |  |  |
| Sex (female) | -.23 | p=.04 |  |  | -.27 | p<.01 |  |  | -.23 | p=.04 |  |  |
| PANSS-R |  |  |  |  | -.39 | p<.001 |  |  | -.43 | p<.001 |  |  |
| Living indepent (ref) |  |  |  |  |  |  |  |  |  |  |  |  |
| With parents/family |  |  |  |  |  |  |  |  | -.27 | p=.11 |  |  |
| Healthcare institution |  |  |  |  |  |  |  |  | .12 | p=.42 |  |  |
| Employed (ref) |  |  |  |  |  |  |  |  |  |  |  |  |
| Unpaid employment |  |  |  |  |  |  |  |  | -.35 | p=.01 |  |  |
| Unemployed |  |  |  |  |  |  |  |  | -.23 | p=.09 |  |  |

| Table 7: linear mixed model (adjusted for age, sex, PANSS-R, living conditions, employment situation) examining if neurocognition predicts the course of the ReQOL | | | | | | | | | | | | |
| --- | --- | --- | --- | --- | --- | --- | --- | --- | --- | --- | --- | --- |
|  | Model adjusted for age, sex | | | | Model adjusted for age, sex, PANSS | | | | Model adjusted for age, sex, PANSS, employment, living conditions | | | |
|  | SE | p-value | Marg. R^2^ | Cond. R^2^ | SE | p-value | Marg. R^2^ | Cond. R^2^ | SE | p-value | Marg. R^2^ | Cond. R^2^ |
| Time | .11 | p<.001 | .03 | .66 | .12 | p<.001 | .17 | .66 | .10 | p<.001 | .20 | .66 |
| Tower of London | .04 | p=.89 |  |  | .03 | p=.87 |  |  | .00 | p=.92 |  |  |
| Time * Tower of London | .03 | p=.24 |  |  | .02 | p=.38 |  |  | .02 | p=.55 |  |  |
| Age | -.05 | p=.38 |  |  | -.06 | p=.26 |  |  | -.09 | p=.15 |  |  |
| Sex (female) | -.23 | p=.04 |  |  | -.26 | p=.01 |  |  | -.23 | p=.04 |  |  |
| PANSS-R |  |  |  |  | -.38 | p<.001 |  |  | -.42 | p<.001 |  |  |
| Living indepent (ref) |  |  |  |  |  |  |  |  |  |  |  |  |
| With parents/family |  |  |  |  |  |  |  |  | -.26 | p=.13 |  |  |
| Healthcare institution |  |  |  |  |  |  |  |  | .11 | p=.47 |  |  |
| Employed (ref) |  |  |  |  |  |  |  |  |  |  |  |  |
| Unpaid employment |  |  |  |  |  |  |  |  | -.35 | p=.01 |  |  |
| Unemployed |  |  |  |  |  |  |  |  | -.24 | p=.08 |  |  |

**Appendix A2**

| Table 8: linear mixed model (adjusted for age, sex, PANSS-R, living conditions, employment situation) examining if neurocognition composite predicts the course of the I.ROC | | | | | | | | | | | | |
| --- | --- | --- | --- | --- | --- | --- | --- | --- | --- | --- | --- | --- |
|  | Model adjusted for age, sex | | | | Model adjusted for age, sex, PANSS | | | | Model adjusted for age, sex, PANSS, employment, living conditions | | | |
|  | SE | p-value | Marg. R^2^ | Cond. R^2^ | SE | p-value | Marg. R^2^ | Cond. R^2^ | SE | p-value | Marg. R^2^ | Cond. R^2^ |
| Time | .09 | p<.01 | .05 | .55 | .09 | p<.001 | .18 | .55 | .08 | p=.02 | .20 | .57 |
| Composite | .14 | p<.01 |  |  | .07 | p=.14 |  |  | .08 | p=.12 |  |  |
| Time * Composite | -.03 | p=.35 |  |  | -.03 | p=.41 |  |  | -.03 | p=.42 |  |  |
| Age | -.05 | p=.35 |  |  | -.09 | p=.11 |  |  | -.07 | p=.30 |  |  |
| Sex (female) | -.19 | p=.09 |  |  | -.23 | p<.05 |  |  | -.20 | p=.06 |  |  |
| PANSS-R |  |  |  |  | -.37 | p<.001 |  |  | -.33 | p<.001 |  |  |
| Living indepent (ref) |  |  |  |  |  |  |  |  |  |  |  |  |
| With parents/family |  |  |  |  |  |  |  |  | -.06 | p=.70 |  |  |
| Healthcare institution |  |  |  |  |  |  |  |  | .04 | p=.80 |  |  |
| Employed (ref) |  |  |  |  |  |  |  |  |  |  |  |  |
| Unpaid employment |  |  |  |  |  |  |  |  | -.22 | p=.12 |  |  |
| Unemployed |  |  |  |  |  |  |  |  | -.42 | p<.01 |  |  |

| Table 9: linear mixed model (adjusted for age, sex, PANSS-R, living conditions, employment situation) examining if Verbal Memory predicts the course of the I.ROC | | | | | | | | | | | | |
| --- | --- | --- | --- | --- | --- | --- | --- | --- | --- | --- | --- | --- |
|  | Model adjusted for age, sex | | | | Model adjusted for age, sex, PANSS | | | | Model adjusted for age, sex, PANSS, employment, living conditions | | | |
|  | SE | p-value | Marg. R^2^ | Cond. R^2^ | SE | p-value | Marg. R^2^ | Cond. R^2^ | SE | p-value | Marg. R^2^ | Cond. R^2^ |
| Time | .08 | p<.01 | .04 | .56 | .09 | p<.01 | .17 | .56 | .08 | p=.02 | .19 | .58 |
| Verbal Memory | .10 | p=.07 |  |  | .04 | p=.40 |  |  | .04 | p=.48 |  |  |
| Time * Verbal Memory | .00 | p=.85 |  |  | .00 | p=.91 |  |  | .00 | p=.87 |  |  |
| Age | -.09 | p=.11 |  |  | -.11 | p=.03 |  |  | -.10 | p=.12 |  |  |
| Sex (female) | -.15 | p=.17 |  |  | -.22 | p=.03 |  |  | -.21 | p=.06 |  |  |
| PANSS-R |  |  |  |  | -.38 | p<.001 |  |  | -.36 | p<.001 |  |  |
| Living indepent (ref) |  |  |  |  |  |  |  |  |  |  |  |  |
| With parents/family |  |  |  |  |  |  |  |  | -.09 | p=.61 |  |  |
| Healthcare institution |  |  |  |  |  |  |  |  | -.04 | p=.81 |  |  |
| Employed (ref) |  |  |  |  |  |  |  |  |  |  |  |  |
| Unpaid employment |  |  |  |  |  |  |  |  | -.24 | p=.09 |  |  |
| Unemployed |  |  |  |  |  |  |  |  | -.35 | p=.01 |  |  |

| Table 10: linear mixed model (adjusted for age, sex, PANSS-R, living conditions, employment situation) examining if Digit Sequencing predicts the course of the I.ROC | | | | | | | | | | | | |
| --- | --- | --- | --- | --- | --- | --- | --- | --- | --- | --- | --- | --- |
|  | Model adjusted for age, sex | | | | Model adjusted for age, sex, PANSS | | | | Model adjusted for age, sex, PANSS, employment, living conditions | | | |
|  | SE | p-value | Marg. R^2^ | Cond. R^2^ | SE | p-value | Marg. R^2^ | Cond. R^2^ | SE | p-value | Marg. R^2^ | Cond. R^2^ |
| Time | .09 | p<.01 | .04 | .56 | .09 | p<.01 | .18 | .57 | .08 | p=.01 | .20 | .58 |
| Digit Sequencing | .11 | p<.01 |  |  | .08 | p=.02 |  |  | .09 | p=.02 |  |  |
| Time * Digit Sequencing | -.05 | p=.14 |  |  | -.05 | p=.14 |  |  | -.05 | p=.12 |  |  |
| Age | -.09 | p=.07 |  |  | -.08 | p=.04 |  |  | -.09 | p=.15 |  |  |
| Sex (female) | -.13 | p=.24 |  |  | -.19 | p=.06 |  |  | -.16 | p=.16 |  |  |
| PANSS-R |  |  |  |  | -.38 | p<.001 |  |  | -.34 | p<.001 |  |  |
| Living indepent (ref) |  |  |  |  |  |  |  |  |  |  |  |  |
| With parents/family |  |  |  |  |  |  |  |  | -.07 | p=.69 |  |  |
| Healthcare institution |  |  |  |  |  |  |  |  | .02 | p=.91 |  |  |
| Employed (ref) |  |  |  |  |  |  |  |  |  |  |  |  |
| Unpaid employment |  |  |  |  |  |  |  |  | -.22 | p=.12 |  |  |
| Unemployed |  |  |  |  |  |  |  |  | -.37 | p<.01 |  |  |

| Table 11: linear mixed model (adjusted for age, sex, PANSS-R, living conditions, employment situation) examining if Token Motor predicts the course of the I.ROC | | | | | | | | | | | | |
| --- | --- | --- | --- | --- | --- | --- | --- | --- | --- | --- | --- | --- |
|  | Model adjusted for age, sex | | | | Model adjusted for age, sex, PANSS | | | | Model adjusted for age, sex, PANSS, employment, living conditions | | | |
|  | SE | p-value | Marg. R^2^ | Cond. R^2^ | SE | p-value | Marg. R^2^ | Cond. R^2^ | SE | p-value | Marg. R^2^ | Cond. R^2^ |
| Time | .09 | p<.01 | .04 | .55 | .09 | p<.01 | .17 | .56 | .08 | p=.02 | .19 | .57 |
| Token Motor | .13 | p=.01 |  |  | .06 | p=.14 |  |  | .08 | p=.10 |  |  |
| Time * Token Motor | -.02 | p=.52 |  |  | -.02 | p=.51 |  |  | -.03 | p=.39 |  |  |
| Age | -.06 | p=.28 |  |  | -.09 | p=.07 |  |  | -.08 | p=.18 |  |  |
| Sex (female) | -.16 | p=.13 |  |  | -.23 | p=.02 |  |  | -.20 | p=.06 |  |  |
| PANSS-R |  |  |  |  | -.38 | p<.001 |  |  | -.34 | p<.001 |  |  |
| Living indepent (ref) |  |  |  |  |  |  |  |  |  |  |  |  |
| With parents/family |  |  |  |  |  |  |  |  | -.09 | p=.59 |  |  |
| Healthcare institution |  |  |  |  |  |  |  |  | .01 | p=.93 |  |  |
| Employed (ref) |  |  |  |  |  |  |  |  |  |  |  |  |
| Unpaid employment |  |  |  |  |  |  |  |  | -.21 | p=.14 |  |  |
| Unemployed |  |  |  |  |  |  |  |  | -.39 | p<.01 |  |  |

| Table 12: linear mixed model (adjusted for age, sex, PANSS-R, living conditions, employment situation) examining if Verbal Fluency predicts the course of the I.ROC | | | | | | | | | | | | |
| --- | --- | --- | --- | --- | --- | --- | --- | --- | --- | --- | --- | --- |
|  | Model adjusted for age, sex | | | | Model adjusted for age, sex, PANSS | | | | Model adjusted for age, sex, PANSS, employment, living conditions | | | |
|  | SE | p-value | Marg. R^2^ | Cond. R^2^ | SE | p-value | Marg. R^2^ | Cond. R^2^ | SE | p-value | Marg. R^2^ | Cond. R^2^ |
| Time | .09 | p<.01 | .03 | .55 | .09 | p<.01 | .17 | .56 | .08 | p=.01 | .19 | .57 |
| Verbal Fluency | .01 | p=.82 |  |  | .00 | p=.83 |  |  | .00 | p=.90 |  |  |
| Time * Verbal Fluency | -.00 | p=.96 |  |  | .00 | p=.94 |  |  | .01 | p=.73 |  |  |
| Age | -.10 | p=.04 |  |  | -.12 | p=.01 |  |  | -.11 | p=.05 |  |  |
| Sex (female) | -.17 | p=.10 |  |  | -.23 | p=.02 |  |  | -.21 | p=.05 |  |  |
| PANSS-R |  |  |  |  | -.38 | p<.001 |  |  | -.35 | p<.001 |  |  |
| Living indepent (ref) |  |  |  |  |  |  |  |  |  |  |  |  |
| With parents/family |  |  |  |  |  |  |  |  | -.09 | p=.59 |  |  |
| Healthcare institution |  |  |  |  |  |  |  |  | -.03 | p=.84 |  |  |
| Employed (ref) |  |  |  |  |  |  |  |  |  |  |  |  |
| Unpaid employment |  |  |  |  |  |  |  |  | -.25 | p=.07 |  |  |
| Unemployed |  |  |  |  |  |  |  |  | -.38 | p<.01 |  |  |

| Table 13: linear mixed model (adjusted for age, sex, PANSS-R, living conditions, employment situation) examining if Symbol Coding predicts the course of the I.ROC | | | | | | | | | | | | |
| --- | --- | --- | --- | --- | --- | --- | --- | --- | --- | --- | --- | --- |
|  | Model adjusted for age, sex | | | | Model adjusted for age, sex, PANSS | | | | Model adjusted for age, sex, PANSS, employment, living conditions | | | |
|  | SE | p-value | Marg. R^2^ | Cond. R^2^ | SE | p-value | Marg. R^2^ | Cond. R^2^ | SE | p-value | Marg. R^2^ | Cond. R^2^ |
| Time | .09 | p<.01 | .05 | .54 | .09 | p<.01 | .18 | .55 | .08 | p=.01 | .18 | .56 |
| Symbol Coding | .12 | p=.02 |  |  | .04 | p=.37 |  |  | .05 | p=.39 |  |  |
| Time * Symbol Coding | -.02 | p=.60 |  |  | -.01 | p=.73 |  |  | .00 | p=.88 |  |  |
| Age | -.08 | p=.14 |  |  | -.12 | p=.02 |  |  | -.11 | p=.09 |  |  |
| Sex (female) | -.20 | p=.07 |  |  | -.24 | p=.02 |  |  | -.21 | p=.05 |  |  |
| PANSS-R |  |  |  |  | -.38 | p<.001 |  |  | -.34 | p<.001 |  |  |
| Living indepent (ref) |  |  |  |  |  |  |  |  |  |  |  |  |
| With parents/family |  |  |  |  |  |  |  |  | -.08 | p=.63 |  |  |
| Healthcare institution |  |  |  |  |  |  |  |  | -.04 | p=.79 |  |  |
| Employed (ref) |  |  |  |  |  |  |  |  |  |  |  |  |
| Unpaid employment |  |  |  |  |  |  |  |  | -.26 | p=.07 |  |  |
| Unemployed |  |  |  |  |  |  |  |  | -.41 | p<.01 |  |  |

| Table 14: linear mixed model (adjusted for age, sex, PANSS-R, living conditions, employment situation) examining if Tower of London predicts the course of the I.ROC | | | | | | | | | | | | |
| --- | --- | --- | --- | --- | --- | --- | --- | --- | --- | --- | --- | --- |
|  | Model adjusted for age, sex | | | | Model adjusted for age, sex, PANSS | | | | Model adjusted for age, sex, PANSS, employment, living conditions | | | |
|  | SE | p-value | Marg. R^2^ | Cond. R^2^ | SE | p-value | Marg. R^2^ | Cond. R^2^ | SE | p-value | Marg. R^2^ | Cond. R^2^ |
| Time | .08 | p<.01 | .04 | .56 | .09 | p<.01 | .18 | .56 | .08 | p=.01 | .20 | .57 |
| Tower of London | .11 | p<.05 |  |  | .09 | p<.05 |  |  | .09 | p=.07 |  |  |
| Time * Tower of London | -.00 | p=.75 |  |  | -.01 | p=.64 |  |  | -.02 | p=.57 |  |  |
| Age | -.11 | p<.05 |  |  | -.11 | p=.02 |  |  | -.10 | p=.10 |  |  |
| Sex (female) | -.18 | p=.10 |  |  | -.22 | p=.03 |  |  | -.20 | p=.07 |  |  |
| PANSS-R |  |  |  |  | -.37 | p<.001 |  |  | -.33 | p<.001 |  |  |
| Living indepent (ref) |  |  |  |  |  |  |  |  |  |  |  |  |
| With parents/family |  |  |  |  |  |  |  |  | .00 | p=.96 |  |  |
| Healthcare institution |  |  |  |  |  |  |  |  | .04 | p=.81 |  |  |
| Employed (ref) |  |  |  |  |  |  |  |  |  |  |  |  |
| Unpaid employment |  |  |  |  |  |  |  |  | -.24 | p=.09 |  |  |
| Unemployed |  |  |  |  |  |  |  |  | -.41 | p<.01 |  |  |
